# Rare and complex diseases in focus: ChatGPT's role in improving diagnosis and treatment

**DOI:** 10.3389/frai.2024.1338433

**Published:** 2024-01-11

**Authors:** Yue Zheng, Xu Sun, Baijie Feng, Kai Kang, Yuqi Yang, Ailin Zhao, Yijun Wu

**Affiliations:** ^1^Cancer Center, West China Hospital, Sichuan University, Chengdu, Sichuan, China; ^2^Department of Hematology, West China Hospital, Sichuan University, Chengdu, Sichuan, China; ^3^West China School of Medicine, Sichuan University, Chengdu, Sichuan, China

**Keywords:** ChatGPT, artificial intelligence, rare and complex diseases, diagnosis, treatment

## Abstract

Rare and complex diseases pose significant challenges to both patients and healthcare providers. These conditions often present with atypical symptoms, making diagnosis and treatment a formidable task. In recent years, artificial intelligence and natural language processing technologies have shown great promise in assisting medical professionals in diagnosing and managing such conditions. This paper explores the role of ChatGPT, an advanced artificial intelligence model, in improving the diagnosis and treatment of rare and complex diseases. By analyzing its potential applications, limitations, and ethical considerations, we demonstrate how ChatGPT can contribute to better patient outcomes and enhance the healthcare system's overall effectiveness.

## 1 Introduction

Rare and complex diseases, often referred to as orphan diseases, affect a relatively small number of individuals but can have devastating effects on those afflicted. Due to the rarity and complexity of these conditions, they are frequently misdiagnosed or remain undiagnosed for extended periods, leading to delayed or inadequate treatment. However, the emergence of artificial intelligence (AI) and natural language processing (NLP) technologies has opened up new possibilities for addressing the diagnostic and therapeutic challenges associated with these diseases (Krishnan et al., 2023). One such example is idiopathic pulmonary arterial hypertension (IPAH), a rare and progressive lung disorder. AI-driven tools have been harnessed to scrutinize medical imaging data, such as echocardiograms, enabling early detection of IPAH and facilitating prompt intervention (Kiely et al., 2019). This demonstrates how the synergy of AI and NLP technologies provides promising solutions for tackling the diagnostic and therapeutic hurdles presented by rare and complex diseases, ultimately enhancing the quality of life for patients.

## 2 Discussion

**Figure 1:**
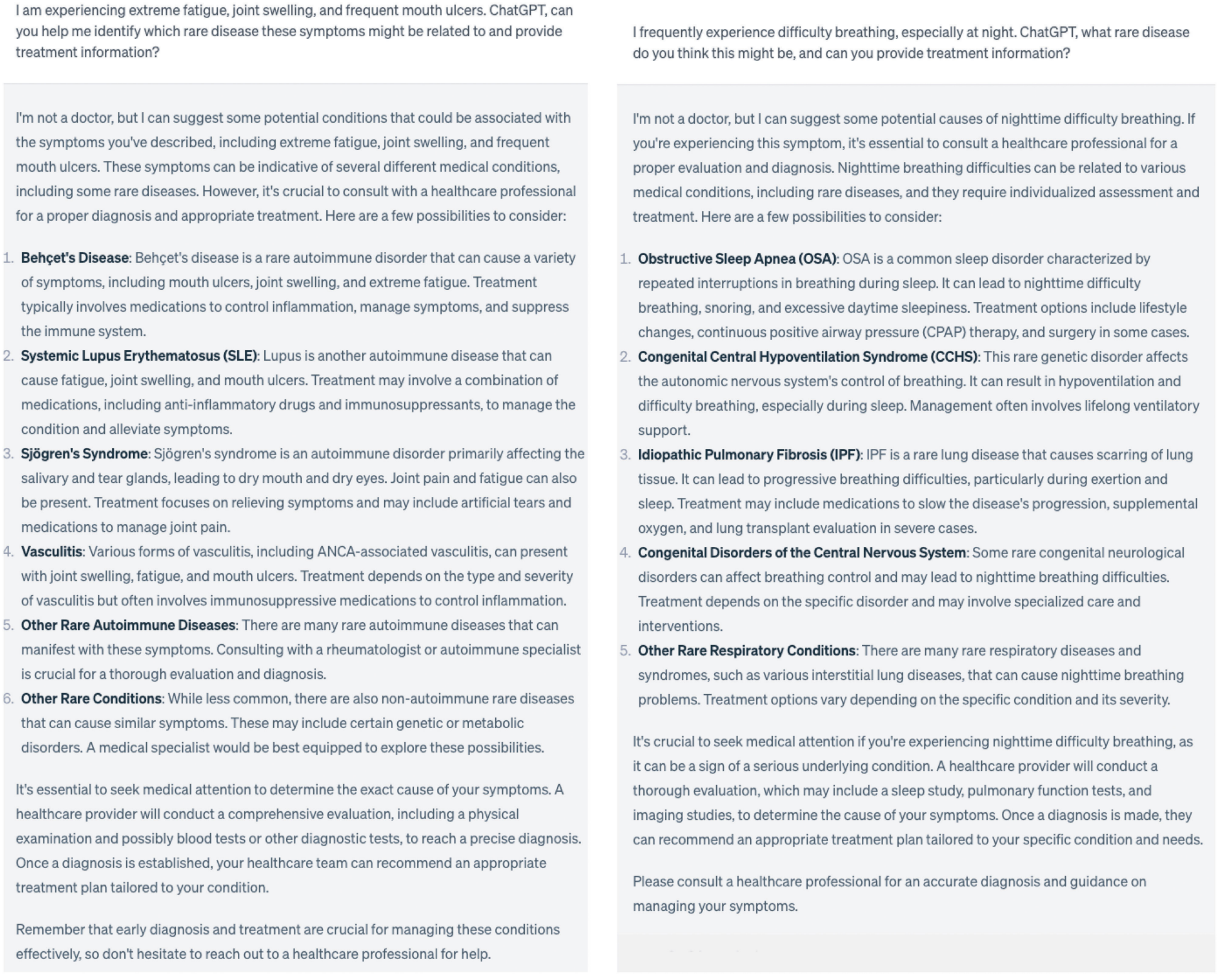
Example of ChatGPT in helping diagnosing and treating rare and complex diseases.

In the realm of healthcare, ChatGPT, as an advanced language model, has demonstrated remarkable potential (Liu et al., 2023; Vaishya et al., 2023). AI and NLP tools empower healthcare professionals to rapidly analyze extensive volumes of medical literature and patient records, possibly aiding in the diagnosis and comprehension of rare diseases like NLP, Klippel-Trenaunay syndrome, Early-Onset Parkinson's Disease, and Rett Syndrome (Table 1). ChatGPT, as a powerful language model, serves as a valuable assistant to physicians and researchers, facilitating access to relevant information, exploration of cutting-edge treatment modalities, and improved communication with patients (Figure 1).

**Table 1:** Example of ChatGPT's possible contributions in helping diagnosing and treating rare and complex diseases.

**Disease**	**Possible contribution of ChatGPT**
Idiopathic pulmonary Arterial hypertension	Early detection through artificial intelligence-driven analysis of medical imaging data
Klippel-trenaunay syndrome	Rapid analysis of extensive medical literature for better understanding
Early-onset Parkinson's disease	Analyzing patient symptoms, even vague or unconventional ones
Rett syndrome	Access to latest research findings and personalized treatment plans

One noteworthy capability of ChatGPT is its ability to analyze patient symptoms, even when they are vague or unconventional. By processing textual descriptions from patients or medical professionals, ChatGPT can generate relevant diagnostic suggestions and highlight potential indicators of rare diseases. Additionally, ChatGPT's proficiency in quickly accessing and summarizing vast amounts of medical literature helps physicians stay abreast of the latest research findings related to rare diseases, potentially leading to more accurate diagnoses (Lapidus, 2023). Additionally, AI technology holds promise in disease prediction and early screening (Au and Yang, 2023; Haver et al., 2023), facilitating the identification of pre-symptomatic signs of rare diseases by analyzing genetic data, biomarkers, and clinical information. This approach expedites early intervention and treatment implementation, potentially improving patient outcomes.

Furthermore, ChatGPT can contribute significantly to personalized treatment plans by considering individual patient data, medical history, and the latest clinical guidelines. It assists in tailoring treatment strategies that account for the unique characteristics of each rare disease case. Moreover, ChatGPT aids in expediting drug discovery for rare diseases by analyzing scientific literature and identifying potential drug candidates or existing medications with repurposing potential, thus reducing the time and cost associated with drug development (Alves et al., 2022).

However, it is important to note that the effectiveness of ChatGPT is contingent on the quality and diversity of the data it has been trained on. Bias in training data can lead to inaccurate recommendations and reinforce healthcare disparities (Aronson, 2023). Besides, ChatGPT's reliance on patterns learned from training data may pose challenges in comprehending the nuanced contextual aspects of individual patient situations. This limitation underscores the necessity for human involvement in interpreting and communicating decisions to patients, ensuring a more accurate and empathetic understanding of their unique circumstances (Tustumi et al., 2023).

It's crucial to emphasize that while ChatGPT provides valuable assistance, it should not replace the expertise of medical professionals. Collaboration between AI and healthcare providers remains essential to ensure patient safety and the provision of quality care (Derevianko et al., 2023). Moreover, in recognizing the pivotal collaboration between AI and human expertise, the realistic scenario involves humans managing and collaborating with chatbots, rather than seeking outright substitution. Human oversight is indispensable for ensuring accuracy, ethical adherence, and a personalized touch in patient interactions. Human intervention plays a crucial role, especially in addressing complex or unconventional cases where ChatGPT's limitations in understanding context, emotions, and unique patient scenarios become apparent.

Ethical concerns surrounding patient data, algorithmic bias, and the responsible use of AI must be addressed to harness the full potential of these technologies in improving rare disease diagnosis and treatment while upholding patient rights and wellbeing (Dave et al., 2023; Liebrenz et al., 2023). This emphasis on ethical considerations ensures that the integration of AI in healthcare aligns with ethical standards and benefits patients without compromising their rights.

Looking ahead to the future, ChatGPT's role is poised to transcend language comprehension and expand into a comprehensive medical model. This evolution entails a multifaceted approach, encompassing text-based interactions, image recognition for assessing physical symptoms, and the capability to analyze uploaded medical images such as rare skin conditions or radiological scans for a holistic diagnosis. In this advanced medical application, ChatGPT would serve as a versatile healthcare assistant, capable of processing both textual and visual data. Patients and healthcare professionals would be able to interact with the model through text, voice, or image inputs, facilitating a more comprehensive understanding of patients' health concerns. For instance, when presented with textual descriptions of symptoms, ChatGPT can offer diagnostic suggestions and potential treatment options. Simultaneously, it can analyze uploaded images to identify visible physical symptoms, such as rare skin diseases or other external manifestations, further enhancing the diagnostic process. Additionally, ChatGPT can process complex medical imaging data, helping healthcare providers in identifying and evaluating intricate conditions, from rare diseases to anomalies detected in radiological scans (Bhayana et al., 2023). This expanded role of ChatGPT in the medical domain holds the promise of improving the accuracy and efficiency of diagnosis and treatment, particularly in the case of rare and complex diseases. However, it is crucial to address ethical and privacy considerations rigorously, ensuring the responsible use of patient data and maintaining a strong collaboration between AI and healthcare professionals. As AI technologies continue to advance, their integration into comprehensive medical models like ChatGPT represents a significant step forward in enhancing healthcare delivery and patient outcomes.

In conclusion, ChatGPT and AI/NLP technologies offer innovative solutions for addressing the diagnostic and therapeutic challenges presented by rare and complex diseases. By leveraging these advancements, healthcare professionals can enhance their ability to diagnose rare diseases promptly and accurately, customize treatments, and accelerate drug discovery. However, it is imperative to address limitations and ethical considerations associated with AI in healthcare, ensuring both patient safety and data privacy. As AI continues to evolve, its role in managing rare diseases is likely to expand, ultimately benefiting patients and advancing our understanding of these complex conditions.

## Data availability statement

The original contributions presented in the study are included in the article/supplementary material, further inquiries can be directed to the corresponding authors.

## Author contributions

YZ: Conceptualization, Investigation, Writing—original draft. XS: Investigation, Writing—original draft. BF: Investigation, Software, Writing—original draft. KK: Investigation, Software, Writing—original draft. YY: Data curation, Validation, Writing—review & editing. AZ: Conceptualization, Funding acquisition, Project administration, Resources, Supervision, Validation, Writing—review & editing. YW: Conceptualization, Funding acquisition, Project administration, Resources, Supervision, Validation, Writing—review & editing.
